# Discovery of ODM-201, a new-generation androgen receptor inhibitor targeting resistance mechanisms to androgen signaling-directed prostate cancer therapies

**DOI:** 10.1038/srep12007

**Published:** 2015-07-03

**Authors:** Anu-Maarit Moilanen, Reetta Riikonen, Riikka Oksala, Laura Ravanti, Eija Aho, Gerd Wohlfahrt, Pirjo S. Nykänen, Olli P. Törmäkangas, Jorma J. Palvimo, Pekka J. Kallio

**Affiliations:** 1 Orion Corporation, Orion Pharma, Finland; 2 Institute of Biomedicine, University of Eastern Finland, Kuopio, Finland

## Abstract

Activation of androgen receptor (AR) is crucial for prostate cancer growth. Remarkably, also castration-resistant prostate cancer (CRPC) is dependent on functional AR, and several mechanisms have been proposed to explain the addiction. Known causes of CRPC include gene amplification and overexpression as well as point mutations of AR. We report here the pharmacological profile of ODM-201, a novel AR inhibitor that showed significant antitumor activity and a favorable safety profile in phase 1/2 studies in men with CRPC. ODM-201 is a full and high-affinity AR antagonist that, similar to second-generation antiandrogens enzalutamide and ARN-509, inhibits testosterone-induced nuclear translocation of AR. Importantly, ODM-201 also blocks the activity of the tested mutant ARs arising in response to antiandrogen therapies, including the F876L mutation that confers resistance to enzalutamide and ARN-509. In addition, ODM-201 reduces the growth of AR-overexpressing VCaP prostate cancer cells both *in vitro* and in a castration-resistant VCaP xenograft model. In contrast to other antiandrogens, ODM-201 shows negligible brain penetrance and does not increase serum testosterone levels in mice. In conclusion, ODM-201 is a potent AR inhibitor that overcomes resistance to AR-targeted therapies by antagonizing both overexpressed and mutated ARs. ODM-201 is currently in a phase 3 trial in CRPC.

Prostate cancer (PC) is the most common cancer and among the three leading causes of cancer deaths in men in the United States^1^ and in Europe^2^. The activation of androgen receptor (AR) signaling is crucial for PC growth at all stages of the disease^3^,^4^,^5^. Despite androgen-deprivation therapy or treatment with the first-generation antiandrogens, most PC patients eventually build up resistance to the treatments and develop a more aggressive form of the disease called castration-resistant prostate cancer (CRPC) that is associated with tumor progression and poor prognosis^6^. For years, docetaxel was the only treatment for CRPC showing a median prolongation of survival of 2.9 months^7^. In recent years, however, new treatment options for CRPC with different mechanisms of action have become available. Many of the new treatments target AR signaling such as CYP17A1 inhibitor abiraterone acetate^8^ and a second-generation AR antagonist enzalutamide^9^,^10^.

CRPC typically arises through mechanisms involving AR, as shown by studies demonstrating the role of autocrine synthesis of androgens and AR protein overexpression in CRPC^11^,^12^,^13^. AR aberrations commonly associated with CRPC include AR amplifications, mutations, and constitutively active AR splice variants^14^,^15^,^16^,^17^,^18^,^19^,^20^. Amplification of the AR gene leading to AR protein overexpression^12^ and mutations in the ligand binding domain (LBD) of AR can make the receptor more sensitive to growth-stimulating effects of low androgen concentrations^21^ and turn antagonist responses to agonistic^22^,^23^,^24^. Certain AR mutations have been linked to the development of resistance to specific antiandrogens, e.g. W741L and T877A mutations have been shown to mediate resistance to first-generation antiandrogens bicalutamide and hydroxyflutamide, respectively^25^,^26^. However, despite the initial response to second-generation AR-targeted agents, resistance develops in nearly all men with metastatic CRPC. Recently, a F876L missense mutation in the LBD of the AR was identified to confer resistance to enzalutamide and ARN-509, an AR antagonist presently in a phase 3 study^27^, by switching these antagonists to agonists^22^,^24^.

In the present study, we describe the discovery and preclinical development of ODM-201, a novel, nonsteroidal, orally active AR inhibitor that inhibits potently androgen binding to AR and androgen-induced nuclear translocation of AR in AR overexpressing cells. Using cells transiently transfected with expression vectors encoding the corresponding mutant AR and an androgen-responsive luciferase reporter gene construct, we show that ODM-201 retains antagonistic properties against clinically relevant AR mutations conferring resistance to antiandrogen therapies. ODM-201 is a full antagonist for the mutant AR(F876L) described to play a role in enzalutamide and ARN-509 resistance, and for mutants AR(W741L) and AR(T877A) shown to mediate resistance to bicalutamide and hydroxyflutamide. ODM-201 shows a more potent antitumor activity in nonclinical models of CRPC, AR-overexpressing VCaP cells, *in vitro* and *in vivo* than other second-generation antiandrogens. Unlike other antiandrogens, ODM-201 exhibits negligible brain penetrance and does not increase serum testosterone levels in mice. These findings have been translated to a promising antitumor activity in CRPC patients, as shown by significant PSA reductions. The PSA response (50% or greater decrease) was present at 86% of chemotherapy-naïve patients at 700 mg twice daily (the highest dose in phase II dose expansion) in the ARADES study (trial registration ID: NCT01429064)^28^. ODM-201 is currently being studied in a large, global phase 3 clinical trial in men with nonmetastatic CRPC (trial registration ID: NCT02200614).

## Results

### Chemical structure of ODM-201

**Figure 1:**
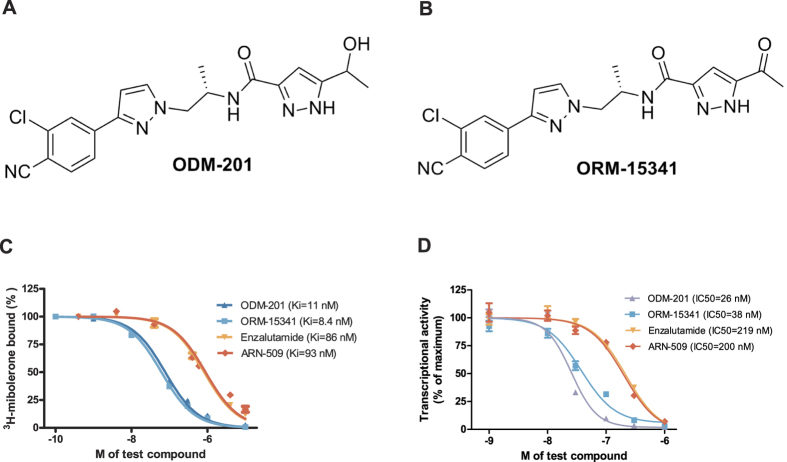
The structures of ODM-201 (A) and its main metabolite ORM-15341 (B). Representative binding affinities of ODM-201, ORM-15341, enzalutamide, and ARN-509 measured in competition with [^3^H]mibolerone using wtAR isolated from rat ventral prostates (**C**). All data points are means of quadruplicates ±SEM. Ki values are presented in parentheses. **D**. Antagonism to wtAR was determined using AR-HEK293 cells treated with ODM-201, ORM-15341, enzalutamide, or ARN-509 together with 0.45 nM testosterone in steroid-depleted medium for 24 hours before luciferase activity measurements. All data points are means of triplicates ±SEM. IC_50_ values are presented in parentheses.

ODM-201 is a synthetic compound discovered by screening campaign using an AR transactivation assay in AR-HEK293 cells and by subsequent medicinal chemistry optimization. ODM-201 and its pharmacologically active main metabolite ORM-15341 are novel and structurally distinct from any known antiandrogens including the second-generation antiandrogens enzalutamide and ARN-509. Figure 1A,B show the chemical structures of ODM-201 and ORM-15341. The synthesis of the compounds is described in Supplementary Data 1.

### High potency of ODM-201 to AR

In competitive AR binding assays, the inhibition constant (Ki) values of ODM-201 and ORM-15341 were 11 and 8 nM, respectively, being clearly lower than those of enzalutamide (86 nM) and ARN-509 (93 nM) (Fig. 1C) that were tested under the same conditions. ODM-201 and its major metabolite ORM-15341 are also potent and full antagonists for human AR (hAR) with IC_50_ values of 26 and 38 nM as shown by transactivation assays in AR-HEK293 cells stably expressing full-length hAR and an androgen-responsive luciferase reporter gene construct. Enzalutamide and ARN-509 have IC_50_ values of 219 and 200 nM, respectively (Fig. 1D). Thus, ODM-201 and its metabolite inhibit AR-mediated transactivation clearly more potently than these clinically tested second-generation antiandrogens.

### ODM-201 does not activate mutant ARs

**Figure 2:**
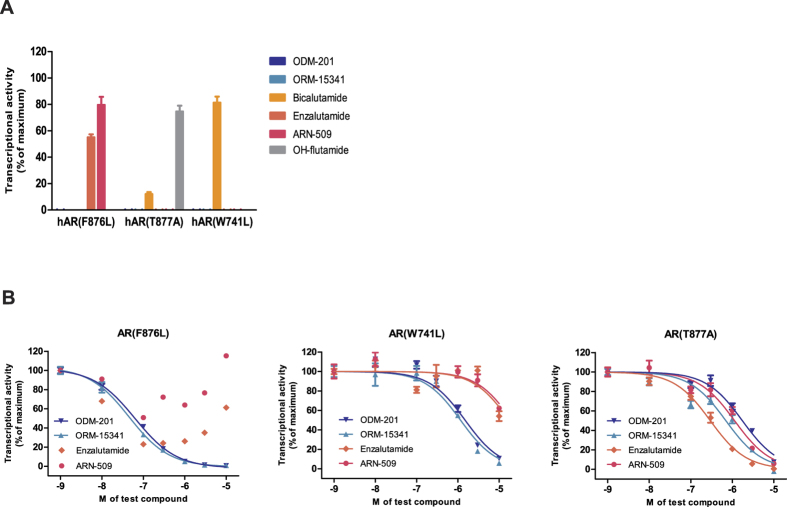
The activation (A) or inhibition (B) of mutant AR(F876L), AR(W741L), and AR(T877A) by ODM-201, ORM-15341, enzalutamide, ARN-509, or bicalutamide and hydroxy (OH)-flutamide (only in the activation assay) was studied in human U2-OS osteosarcoma cells transiently transfected with expression vectors encoding the corresponding mutant AR and an androgen-responsive luciferase reporter gene construct. For assays, steroid-depleted medium was used and luciferase activity was measured after 24 hours. All data points are means of triplicates +SEM. **A**. Bars represent relative transcriptional activity as percentage of control (testosterone or DHT set as 100%). **B**. Test compounds were added together with 0.6 nM testosterone (F876L and T877A) or 10 nM DHT (W741L). IC_50_ values are presented in Table 1.

Emergence of mutations in AR has been suggested to drive resistance to antiandrogen therapies. The effects of antiandrogens on mutant AR(F876L), AR(W741L), and AR(T877A) were studied in transactivation assays in human U2-OS osteosarcoma cells transiently transfected with expression vectors encoding the corresponding mutant AR and an androgen-responsive luciferase reporter gene construct. The F876L substitution in AR switched enzalutamide and ARN-509 from antagonists to agonists, whereas bicalutamide was agonistic for AR(W741L) mutation (Fig. 2A, Supplementary Fig. S1), as previously reported^22^,^23^,^24^,^26^,^29^. Of the tested antiandrogens, only ODM-201 and its main metabolite ORM-15341 functioned as full antagonists for all tested mutant ARs (Fig. 2B). Data on antagonism of the tested antiandrogens with wtAR and mutated ARs are summarized in Table 1.

### Inhibition of nuclear translocation of AR by ODM-201

**Figure 3:**
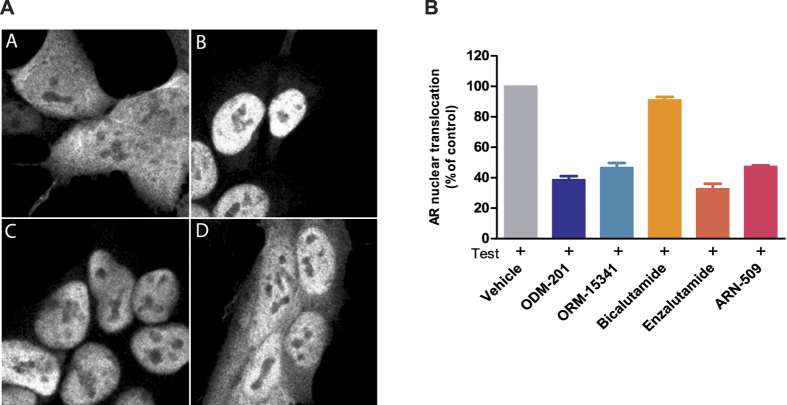
The inhibition of the nuclear translocation of AR. **A**. Representative confocal microscopic images of HS-HEK293 cells treated with DMSO (**A**), testosterone (**B**), testosterone combined with bicalutamide (**C**), or testosterone combined with ODM-201 (**D**). The concentrations of testosterone and test compounds were 0.3 nM and 1.0 μM, respectively. The images are cropped from photographs taken at magnification x63. **B**. AR overexpressing HS-HEK293 cells were treated with 0.3 μM of ODM-201, ORM-15341, bicalutamide, enzalutamide, or ARN-509 with 0.3 nM testosterone in steroid-depleted medium for 4 hours, immunolabeled with an AR antibody conjugated with Alexa Fluor^®^ 488, and imaged with Cellomics ArrayScan VTI HCS reader. Bars represent AR nuclear localization as percentage of the control (testosterone). All data points are means of triplicates +SEM. Test = testosterone.

To investigate the effect of antiandrogens on the subcellular localization of AR, immunocytochemical labeling with an anti-AR antibody in AR overexpressing HS-HEK293 cells was used. As shown in Fig. 3A, AR was predominantly cytoplasmic in the absence of androgen, and exposure to testosterone markedly increased the nuclear-cytoplasmic ratio of AR immunofluorescence intensity, indicating the movement of AR from the cytoplasm to the nucleus. In the presence of bicalutamide, AR was largely nuclear, indicating that bicalutamide failed to block the testosterone-induced nuclear translocation of AR. In contrast, in the presence of ODM-201, ORM-15341, enzalutamide, or ARN-509, AR was predominantly cytoplasmic, showing that these antiandrogens inhibit the androgen-induced nuclear translocation of overexpressed AR to same extent (Fig. 3A,B). Corresponding results were obtained also with an AR-overexpressing PC cell line (LNCaP) (Supplementary Fig. S2).

### ODM-201 inhibits VCaP cell proliferation *in vitro* and tumor growth in a castration-resistant VCaP xenograft model *in vivo*

**Figure 4:**
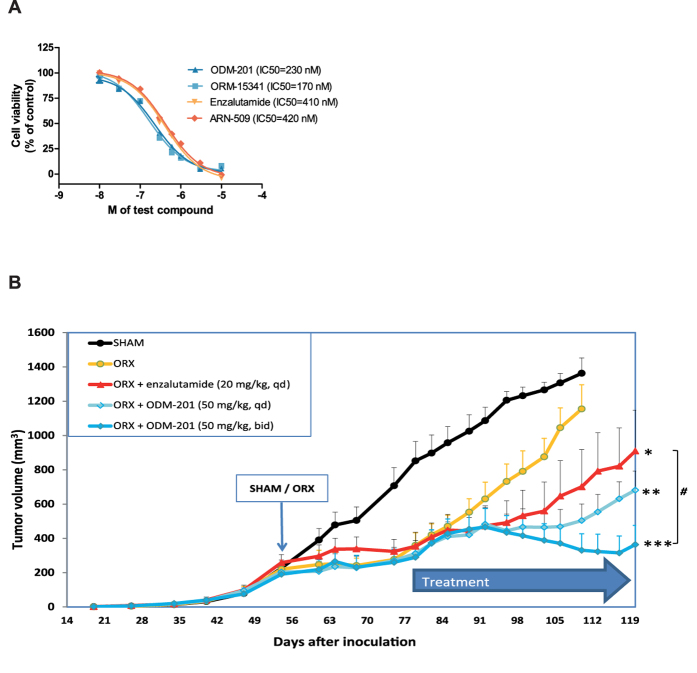
Growth inhibition of VCaP cells in *in vitro* and *in vivo* CRPC settings. **A**. VCaP prostate cancer cells were treated with a submaximal concentration of mibolerone (0.1 nM) and different concentrations of test compounds in steroid-depleted medium, and cell growth was measured after 4 days of incubation using a WST-1 cell proliferation assay. The representative graph of the inhibitory effects of test compounds on the viability of VCaP cells is shown. All data points are means of quadruplicates ±SEM. IC_50_ values are presented in parentheses. **B**. Mean tumor volumes (mm^3^, +SEM) in castrated (ORX) nude mice with subcutaneous VCaP tumors after the oral treatment with ODM-201 (50 mg/kg, once or twice daily) or enzalutamide (20 mg/kg, once daily) for 37 days (n = 6−11). SHAM, sham-operated. ***p < 0.001, **p < 0.01, *p < 0.05 vs. ORX; ^#^p < 0.05 vs. enzalutamide.

To study the antiproliferative properties of ODM-201 and ORM-15341, we used the VCaP cell line originally derived from a bone metastasis of a CRPC patient. The VCaP cell line is characterized with endogenous AR gene amplification and AR overexpression^30^, typical for CRPC. When grown with a submaximal concentration of mibolerone, a synthetic androgen, ODM-201 and ORM-15341 suppressed androgen-induced cell proliferation more efficaciously than enzalutamide or ARN-509, IC_50_ values being 230 and 170 nM for ODM-201 and ORM-15341 vs. 410 and 420 nM for enzalutamide and ARN-509 (Fig. 4A). ODM-201 had no effect on the viability of AR-negative cell lines tested, DU-145 prostate cancer cells and H1581 lung cancer cells (Supplementary Fig. S3) confirming that the antiproliferative properties of ODM-201 and ORM-15341 are specific to AR-dependent PC cells.

To elucidate the *in vivo* efficacy of ODM-201 in a CRPC mouse model, castrated male nude mice with subcutaneously injected VCaP cells were treated orally with ODM-201 (50 mg/kg) once (qd) or twice daily (bid), or with enzalutamide (20 mg/kg, qd) for 37 days. The dose for enzalutamide was selected based on previously published *in vivo* studies^9^ and our pharmacokinetic (PK) analyses which revealed that in mice the systemic exposure (AUC_0–24_) for this dose of enzalutamide was 2.5 times higher than that for ODM-201 (50 mg/kg, bid). Moreover, enzalutamide exhibited a long plasma half-life (18.3 hours) while the half-life of ODM-201 in mice was not optimal (1.6 hours) supporting once daily dosing for enzalutamide and higher dose and more frequent dosing for ODM-201. PK data for enzalutamide and ODM-201 are presented in Supplementary Table S1.

ODM-201 showed a significant antitumor activity with both doses, 50 mg/kg twice daily being more efficacious compared to castrated, untreated mice (p < 0.001) or enzalutamide (p = 0.0245) (Fig. 4B), which also showed inhibition of tumor growth (p < 0.05) vs. castrated, untreated mice. Further, there was no sign of treatment-related toxicities; the body weights of mice treated with ODM-201 twice daily did not decrease significantly during the treatment (Supplementary Table S2).

### ODM-201 does not increase serum testosterone levels or penetrate the blood-brain barrier

**Figure 5:**
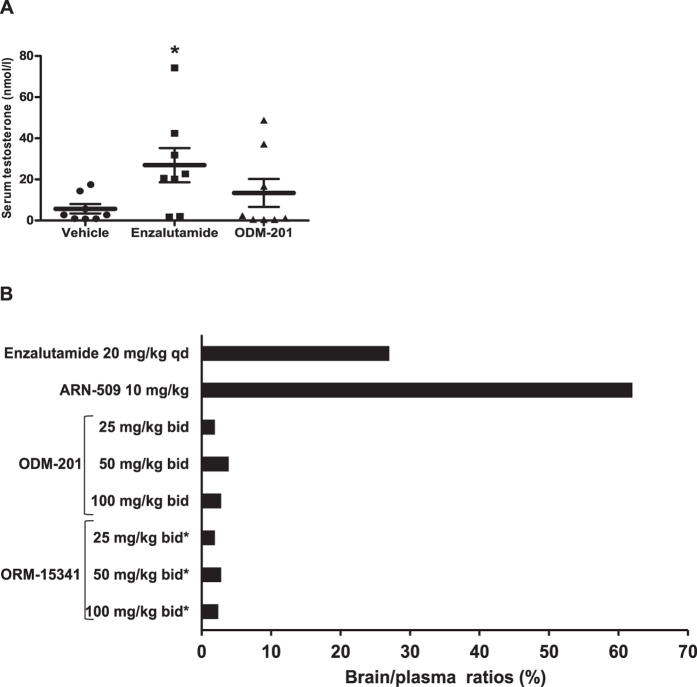
Regulation of testosterone levels. **A**. Serum testosterone levels (nmol/L, ±SEM) of individual intact nude mice bearing orthotopic VCaP tumors after the oral treatment with vehicle, enzalutamide (20 mg/kg, qd), or ODM-201 (50 mg/kg, bid) for 3 weeks (n = 8). *p < 0.05 vs. vehicle. **B**. Brain penetrance. Brain/plasma ratios (%) were calculated based on plasma and brain exposures (AUC_0–24_) after the oral dosing of ODM-201 (25, 50, or 100 mg/kg, bid for 7 days), enzalutamide (20 mg/kg, qd for 7 days) (n = 5), or ARN-509 (a single dose of 10 mg/kg) (n = 3). Data represent mean brain/plasma ratios. *Brain/plasma ratios of ORM-15341 were evaluated in mice treated with different concentrations of ODM-201. Qd = once daily, bid = twice daily.

Serum testosterone levels can increase due to central nervous system stimulation of luteinizing hormone (LH) signaling by the antiandrogen therapy^31^. In an experiment where orthotopic VCaP tumor-bearing intact nude mice were treated orally with enzalutamide (20 mg/kg, qd) or ODM-201 (50 mg/kg,bid) for 3 weeks, enzalutamide increased significantly serum testosterone concentrations (p < 0.05), whereas ODM-201 did not affect the testosterone levels in serum (Fig. 5A). Also in this xenograft model, ODM-201 inhibited tumor growth significantly compared to the control group (Supplementary Fig. S4).

To further evaluate the androgen feedback loop at the hypothalamic-pituitary-gonadal axis, the ability of ODM-201, ORM-15341, enzalutamide, or ARN-509 to enter the brain was evaluated in a series of PK studies. Test compound concentrations (including the ORM-15341 metabolite of ODM-201) were determined in mouse plasma and brain homogenates after 7 days of oral dosing of ODM-201 (25, 50, and 100 mg/kg, all bid) or enzalutamide (20 mg/kg, qd). A single dose of ARN-509 (10 mg/kg) was enough to demonstrate the high penetrance of ARN-509 to the brain, the brain/plasma ratio being 62%. Brain/plasma ratios for different concentrations of ODM-201 and ORM-15341 were very low, 1.9–3.9% and 1.9–2.8%, respectively, with no dose-response, whereas enzalutamide showed marked brain penetrance with the brain/plasma ratio of 27% (Fig. 5B). The very low access of [^14^C]ODM-201 and its metabolites into the brain was confirmed with a quantitative whole-body autoradiography (QWBA) in rat *(data not shown)*.

## Discussion

Here, we describe a novel AR inhibitor ODM-201 which efficiently blocks AR signaling by binding to AR with superior affinity and impairing the nuclear translocation of the overexpressed AR. ODM-201 and its pharmacologically active main metabolite ORM-15341 are structurally distinct from any known antiandrogens including the second-generation antiandrogens enzalutamide and ARN-509. Since the diversity of chemical scaffolds in clinically used antiandrogens has thus far been narrow, new structures are of interest as they might have properties that differ from current antiandrogens.

It is now understood that PC is androgen-sensitive not only during the early stages of the disease but successful treatment with novel AR-targeting therapies indicates that also CRPC remains androgen-sensitive^8^,^10^,^32^. AR mutations are rare in early stage PC before endocrine treatment but are frequent in CRPC^18^. The commonly-found AR mutations in CRPC alter the structure of the ligand binding pocket and broaden the ligand binding specificity of AR, allowing other steroid hormones, corticosteroids, and antiandrogens to activate AR^25^. ODM-201 and its main metabolite were full antagonists to all studied AR mutations shown to drive resistance to both first- and second-generation antiandrogen therapies. Notably, in contrast to enzalutamide and ARN-509^29^, ODM-201 and ORM-15341 both fully antagonized the recently reported AR mutation AR(F876L). This mutation has been detected in plasma DNA from men with CRPC treated with ARN-509, strongly suggesting the F876L mutation to function as a driver of acquired resistance to ARN-509^23^. Based on the *in vitro* data of Joseph *et al.*, 2013 and Balbas *et al.*, 2013, it is likely that the F876L mutation arises also in patients treated with enzalutamide. Further, ODM-201 and its main metabolite functioned as antagonists with the AR mutants AR(T877A) (found in the LNCaP PC cell line) and AR(W741L) conferring the activation of AR-mediated transcription by hydroxyflutamide and bicalutamide. Thus, ODM-201 functions as an antagonist to all well-known AR mutations appearing in response to antiandrogen therapies. Our data support further investigations of clinical responses to ODM-201 in e.g. enzalutamide-resistant CRPC patients.

In VCaP cells containing endogenous AR gene amplification and overexpressing AR, ODM-201 and ORM-15341 inhibited cell proliferation more efficiently than enzalutamide or ARN-509. *In vivo*, ODM-201 showed a significant antitumor activity in a VCaP xenograft model generated to mimic CRPC. In this model, castration of the mice stabilizes tumor growth at first, but after a few weeks, tumors start to grow again (the castration-resistant stage), and at that time treatment with ODM-201 and enzalutamide is initiated. Importantly, bicalutamide (studied at 20 mg/kg, per os) does not have any antitumor activity in this model (*data not shown*). ODM-201 at 50 mg/kg (bid, per os) showed a more potent antitumor activity than enzalutamide (20 mg/kg, qd, per os), although the systemic exposure for enzalutamide was higher than that for ODM-201.

Most of the tumors in the castration-resistant state of the PC still depend on AR for growth and increased androgen levels have been linked to the progression of CRPC^11^,^33^,^34^,^35^. Enzalutamide (20 mg/kg, qd, per os) was shown to increase testosterone levels in mice, which is in accordance with reports of enzalutamide-treated CRPC patients showing increased levels of testosterone in plasma and bone marrow^36^ and increased levels of testosterone and estradiol in serum^37^. Importantly, the data presented here show that ODM-201 does not penetrate the blood-brain barrier and does not increase serum testosterone levels suggesting that it does not stimulate production of androgens via the hypothalamic-pituitary-gonadal axis. These results are in concert with the findings in ODM-201-treated patients, the serum testosterone levels of whom were maintained at castrate levels^28^.

The tendency to induce seizures has been recognized as an obstacle in discovery and development of the second-generation antiandrogens^38^. GABA-A inhibition is a common off-target activity of approved and next-generation AR antagonists potentially explaining these side effects^39^. Seizures have been reported in three (2%) of 140 CRPC patients receiving higher doses of enzalutamide in a phase 1/2 trial^40^, with seizures also reported in seven (1%) of 800 patients in a phase 3 trial^10^. Our PK studies in mice confirmed the ability of enzalutamide and ARN-509 to penetrate the blood-brain barrier, as previously reported^27^, whereas ODM-201 and ORM-15341 showed negligible brain penetrance. The very low concentrations of ODM-201 and ORM-15341 found in the brain were likely to derive from the blood sustained in the vessels when brains were homogenized. The results suggest that ODM-201 has a low risk of inducing seizures in CRPC patients, as supported by clinical data^28^.

In summary, ODM-201 is a high-activity, next-generation AR inhibitor, which antagonizes AR mutants AR(F876L), AR(W741L), and AR(T877A) known to mediate resistance to first- and second-generation antiandrogens. ODM-201 also functions as an antagonist in AR overexpressing cells and impairs nuclear translocation of the receptor. In nonclinical *in vitro* and *in vivo* models of CRPC, ODM-201 is more efficacious than other tested antiandrogens and it does not stimulate androgen feedback loop at the hypothalamic-pituitary-gonadal axis. Taken together, these results indicate that ODM-201 exhibits unique properties that may offer advantages for the treatment of CRPC over the first- and second-generation antiandrogens. These nonclinical findings have been translated to significant antitumor activity and a good tolerability and safety profile observed in phase 1 and 2 clinical trials in men with metastatic CRPC^28^. In these trials, PSA response (50% or greater decrease) was present at 86% of chemotherapy-naïve patients at 700 mg twice daily dose.

## Methods

### Cell lines

U2-OS cells were obtained from Dr. Piia Aarnisalo (University of Helsinki, Finland, 2002) and HEK293 cells from American Type Cell Culture (ATCC, 2001). HEK293 cells stably expressing wtAR (AR-HEK293 cells) were created by transfecting HEK293 cells with an expression vector encoding full-length hAR (pSG5-hAR) and an androgen-responsive reporter gene construct (pcDNA3.1/GRE2-TK-Luc). A cell clone of AR-HEK293 having over 5-fold overexpression of AR (HS-HEK293, *data not shown*) and AR-overexpressing LNCaP cells^41^ (LN-AR-C cells, obtained from Prof. Tapio Visakorpi, University of Tampere, 2011) were used in AR nuclear translocation assays. VCaP cells were obtained in 2006 from ATCC and were last tested and authenticated by short-tandem repeat analysis in January 2014. DU-145 cells were obtained from ATCC in 2008 and H1581 cells in 2011.

AR-HEK293, HS-HEK293, U2-OS, and DU-145 cells were cultured in DMEM medium and VCaP and LN-AR-C cells in RPMI-1640 medium. H1581 cells were cultured in a mixture (1:1) of DMEM and F12 Nutrient mixture. All media were supplemented with 10% fetal bovine serum (FBS), 100 UI/mL penicillin, 100 μg/mL streptomycin, and 2 mM (AR-HEK293, HS-HEK293, and U2-OS) or 4 mM (VCaP, DU-145, LN-AR-C, and H1581) GlutaMAX. For maintaining the selection, medium was supplemented with geneticin (50 mg/ml, LN-AR-C cells) or with hygromycin and geneticin (both 50 mg/ml, HS-HEK cells). For *in vitro* assays, corresponding phenol red-free media supplemented with steroid-depleted FBS were used. All cell culture reagents were purchased from Gibco. Cells were grown in a humidified incubator with 5% CO_2_ at 37 °C.

### Compounds

Testosterone was purchased from Fluka, mibolerone and [^3^H]mibolerone from Perkin Elmer, and DHT from Sigma. Bicalutamide was extracted from pellets (AstraZeneca), and ODM-201 and ORM-15341 were synthetized by Orion Pharma.

### AR binding affinity

AR binding affinities of test compounds were studied in cytosolic lysates obtained from ventral prostates of castrated rats (HsdHan:WIST from Harlan, Netherlands) by a competition binding assay as previously described^42^. Fresh prostates were minced and homogenized with Buffer A containing protease inhibitors (Roche). The homogenates were centrifuged and the resultant supernatants were treated with a dextran-coated charcoal solution to remove endogenous steroids. The dissociation constant of the radio ligand [^3^H]mibolerone for isolated rat ARs was determined in a saturation binding experiment as previously described^43^. For the determination of Ki values, prostate cytosol preparations and 1 nM [^3^H]mibolerone were incubated with increasing concentrations of test compounds overnight. After the incubation, bound and free steroids were separated by treatment with 100 μL of dextran-coated charcoal suspension. Bound radioactivity was determined by counting 100 μL of supernatant fraction in 200 μL of scintillation fluid (OptiPhase SuperMix, PerkinElmer) using a microbeta counter (1450 MicroBeta Trilux, Liquid Scintillation & Luminescence Counter, Wallac). All procedures were carried out at 0–4 °C.

### Antagonism of ODM-201

Functional activity and potency of antiandrogens to hAR were determined in AR-HEK293 cells. The cells were treated with test compounds and 0.45 nM testosterone in steroid-free assay medium supplemented with 2 nM GlutaMAX and 25 mM HEPES. After 24 hours at 37 °C with 5% CO_2,_ cells were lysed and luciferase activity was measured with a Centro LB 960 microplate luminometer (Berthold Technologies) using a luciferase assay system (Promega Corporation) according to manufacturer’s instructions.

### Mutant AR studies

Human U2-OS osteosarcoma cells were transiently transfected with an androgen-responsive reporter gene construct (pGV5-basic-GRE-hiv-luc) and expression vectors encoding AR mutants AR(F876L), AR(T877A), or AR(W741L) (pSG5-hAR-F876L, pSG5-hAR-T877A, or pSG5-hAR-W741L) using Lipofectamin^TM^2000 (Invitrogen). The construction of the mutant AR expression vectors was done as previously described^44^. For one well in a 96-well plate, 190 ng of reporter construct DNA and 10 ng of receptor construct DNA were diluted in Opti-MEM^®^ (Gibco). Cells were treated with increasing concentrations of the test compounds in the absence or presence of a reference agonist inducing a submaximal reporter gene activation (0.6 nM testosterone in case of T877A and F876L, and 10 nM DHT in case of W741L) in steroid-free assay medium and incubated for 24 hours. Luciferase activity was measured as described above.

### AR nuclear translocation

AR overexpressing HS-HEK293 cells immunolabeled with an AR-antibody were imaged either with a high-content screening (HCS) reader (Cellomics ArrayScan HCS VTI reader, Thermo) or with a confocal microscope (LSM780, Zeiss). HS-HEK293 cells in steroid-free assay medium were plated on poly-D-lysine coated microplates (BD) (HCS reader) or on coverslips (confocal imaging). After a 48-hour incubation, the cells were treated with 0.3 (HCS reader) or 1 μM (confocal imaging) of test compounds together with 0.3 nM testosterone for 5 hours. After fixation with 3.7% PFA, the cells were washed with phosphate-buffered saline (PBS), permealized with 0.1% Triton X-100 (Sigma), and treated with 3% BSA in PBS to block unspecific staining. For HCS reader, cells were incubated with polyclonal AR antibody conjugated with Alexa Fluor^®^ 488 (N20, Santa Cruz, dilution 1:50). Cells were washed, DNA was labeled with DAPI (Sigma, 1 μg//mL), and images were analyzed with a NucTrans. V3 assay algorithm (Thermo). For confocal imaging, polyclonal AR antibody (N20, Santa Cruz, dilution 1:300) was used as a primary and Alexa Fluor^®^ 546 anti-rabbit IgG (LifeTechnologies, dilution 1:800) as a secondary antibody. Coverslips were mounted with Vectashield containing DAPI (Vector Laboratories). AR overexpressing LN-AR-C cells were treated with 3 μM of test compounds together with 0.3 nM testosterone for 4 hours, immunolabeled with the AR-antibody (N-20, Santa Cruz, dilution 1:75) and a secondary antibody (goat anti-rabbit IgG DyLight 488, Abcam, dilution 1:200) and imaged with the HCS reader.

### VCaP proliferation assay

VCaP cells were treated with a submaximal concentration of mibolerone (0.1 nM) and increasing concentrations of test compounds in steroid-free assay medium supplemented with 4 mM GlutaMAX. After a 4-day incubation with the compounds, cell viability was measured using a WST-1 cell proliferation assay (Roche), according to manufacturer’s instructions. To rule out non-AR –mediated toxicity, AR-negative PC cells (DU-145) and lung cancer cells (H1581) were treated with an increasing concentration of ODM-201, and cell viability was measured as described above.

### The castration-resistant VCaP xenograft experiment

All animal studies were conducted in accordance with EU legislation^45^, and approved by the Finnish Animal Experiment Board.

BALB/c nude male mice (7 weeks old from Charles River, Germany) were subcutaneously injected with 2 million VCaP cells in 100 μL of RPMI-1640 medium and Matrigel (BD) (1:1). Tumor growth was monitored twice weekly by caliper measurements. The volume of the tumor was calculated according to the formula W^2^ × L/2 (mm^3^), where W is the shorter and L the longer diameter of the tumor. When the average tumor volume reached ~200 mm^3^, mice were castrated or SHAM-operated under Avertin anesthesia. Oral treatments with two doses of ODM-201 (50 mg/kg, qd or bid), enzalutamide (20 mg/kg, qd), or vehicle were initiated upon tumor regrowth (when average tumor volumes were ~400 mm^3^) and were continued for 37 days. For all *in vivo* studies, Macrocol^®^ (Merck) + propylene glycol +5% glucose (50:30:20, v/v/v) was used as a vehicle.

### Serum testosterone

One million VCaP cells in 20 μL of RPMI-1640 medium were orthotopically injected into the dorsal prostate lobes of athymic nude male mice (7 weeks old from Harlan Winkelman, France). Tumor growth was followed by measuring serum PSA (ProStatus™ PSA EQM Kits, Wallac, Finland). Oral treatments were initiated 4 weeks post-inoculation, when mean serum PSA values were approximately 5.5 ug/L. ODM-201 (50 mg/kg, bid), enzalutamide (20 mg/kg, qd), or vehicle were administered for 3 weeks. At autopsy, blood samples were collected by heart puncture, clotted overnight at +4 °C, and centrifuged. For serum testosterone determination, 25-μL aliquots were extracted twice with 2 mL of diethyl ether and evaporated under nitrogen to dryness. The residues were reconstituted in PBS and measured using a standard radioimmunoassay as described previously^46^.

### Pharmacokinetic studies in mice

In the PK studies analyzing the penetration of test compounds to the brain, nude male mice (BALB/c or Balb/cOlaHsd from Charles River Laboratories and Harlan, Netherlands, 8-9 weeks of age) were orally dosed for 7 days with 25, 50, or 100 mg/kg of ODM-201 twice daily (n = 5) or with 20 mg/kg enzalutamide once daily (n = 4), or with a single oral dose of ARN-509 (10 mg/kg) (n = 3). Control mice received vehicle. Blood samples were collected into K_2_EDTA tubes by cardiac puncture under CO_2_ anesthesia and plasma was separated by centrifugation. Brain samples (without olfactory bulbs and medulla oblongata) from each group and time point were pooled and homogenized before the analysis. Concentrations of ODM-201 and ORM-15341 in mouse plasma and brain were determined by liquid chromatography-tandem mass spectrometry (LC-MS/MS) method at Charles River, UK, with the lower limit of quantification (LLOQ) being in plasma 1.00 ng/mL for both ODM-201 and ORM-15341 and in brain 4.00 ng/g for ODM-201 and 10.00 ng/g for ORM-15341. Enzalutamide and ARN-509 concentrations were determined by LC-MS/MS method at Orion Pharma (LLOQ for enzalutamide was 1.00 ng/mL in plasma and 5.00 ng/g in brain, and for ARN-509 0.250 ng/mL in plasma and 10.0 ng/g in brain). Plasma and brain concentration vs. time were evaluated by noncompartmental analysis using WinNonlin^®^ Professional v. 5.2 software (Pharsight Corporation). Brain/plasma ratios were calculated based on AUC_0–24_ values for plasma and brain.

### Data analysis and statistical methods

If not otherwise stated, *in vitro* data were analyzed with GraphPad Prism 5 software (version 5.02) to obtain Ki and IC_50_ values. For the VCaP xenograft experiment, mean tumor volumes were calculated for each treatment group. A repeated measure ANOVA (RMANOVA) was used as a statistical method to analyze tumor volume changes over the treatment time. With regard to the serum testosterone levels, the differences between groups were analyzed using Wilcoxon rank sum test.

## Additional Information

**How to cite this article**: Moilanen, A.-M. *et al.* Discovery of ODM-201, a new-generation androgen receptor inhibitor targeting resistance mechanisms to androgen signaling-directed prostate cancer therapies. *Sci. Rep.* **5**, 12007; doi: 10.1038/srep12007 (2015).

## Supplementary Material

Supplementary Information

## Figures and Tables

**Table 1:** Inhibition (IC_50_ values) of wtAR and mutant AR(F876L), AR(W741L), and AR(T877A) by ODM-201, ORM-15341, enzalutamide, and ARN-509.

Compound	wtAR IC_50_ (nM)	AR(F876L) IC_50_ (nM)	AR(W741L) IC_50_ (nM)	AR(T877A) IC_50_ (nM)
ODM-201	65	66	1500	1782
ORM-15341	25	51	1160	700
Enzalutamide	155	Agonist	>10 000	296
ARN-509	168	Agonist	>10 000	1130
