# Supplementary material for: Discovery of ODM-201, a new-generation androgen receptor inhibitor targeting resistance mechanisms to androgen signaling-directed prostate cancer therapies
Source: Sci Rep. 2015 Jul 3;5:12007. doi: 10.1038/srep12007 (PMC4490394; doi:10.1038/srep12007)
Supplement: Supplementary Information [file srep12007-s1.pdf]

SUPPLEMENTARY INFORMATION

Discovery of ODM-201, a new-generation androgen receptor inhibitor targeting resistance mechanisms to androgen signaling-directed prostate cancer therapies

Anu-Maarit Moilanen[1], Reetta Riikonen[1], Riikka Oksala[1], Laura Ravanti[1], Eija Aho[1], Gerd Wohlfahrt[1], Pirjo S. Nykänen[1], Olli P. Törmäkangas[1], Jorma J. Palvimo[2], Pekka J. Kallio[1, *]

Supplementary Figure S1

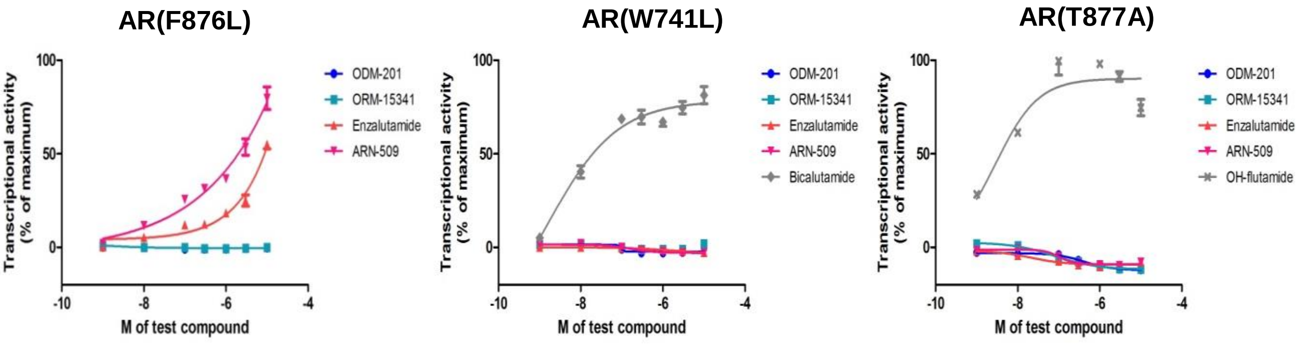

Activation of mutant AR(F876L), AR(W741L), and AR(T877A) by different antiandrogens. Representative graphs of transactivation of mutant ARs by ODM-201, ORM-15341, enzalutamide, ARN-509, bicalutamide (AR(W741L)), or OH-flutamide (AR(T877A)) in human U2-OS osteosarcoma cells transiently transfected with expression vectors encoding the corresponding mutant AR and an androgen-responsive luciferase reporter gene construct. For assays, steroid-depleted medium was used and luciferase activity was measured after 24 hours. All data points are means of triplicates +SEM.

Supplementary Figure S2

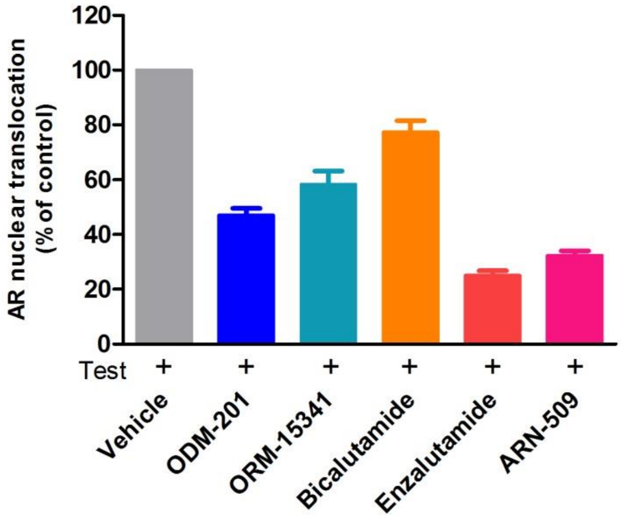

The inhibition of the nuclear translocation of AR by different antiandrogens in AR overexpressing LNCaP cells. AR overexpressing LNCaP cells were treated with 3 µM of ODM-201, ORM-15341, bicalutamide, enzalutamide, or ARN-509 with 0.3 nM testosterone in steroid-depleted medium for 4 hours, immunolabeled with anti-AR primary antibody (N-20, Santa cruz) and secondary antibody conjugated with DyLight 488 (Abcam), and imaged with Cellomics ArrayScan VTI HCS reader. Bars represent AR nuclear localization as percentage of the control (testosterone). All data points are means of triplicates +SEM. Test, testosterone.

Supplementary Figure S3

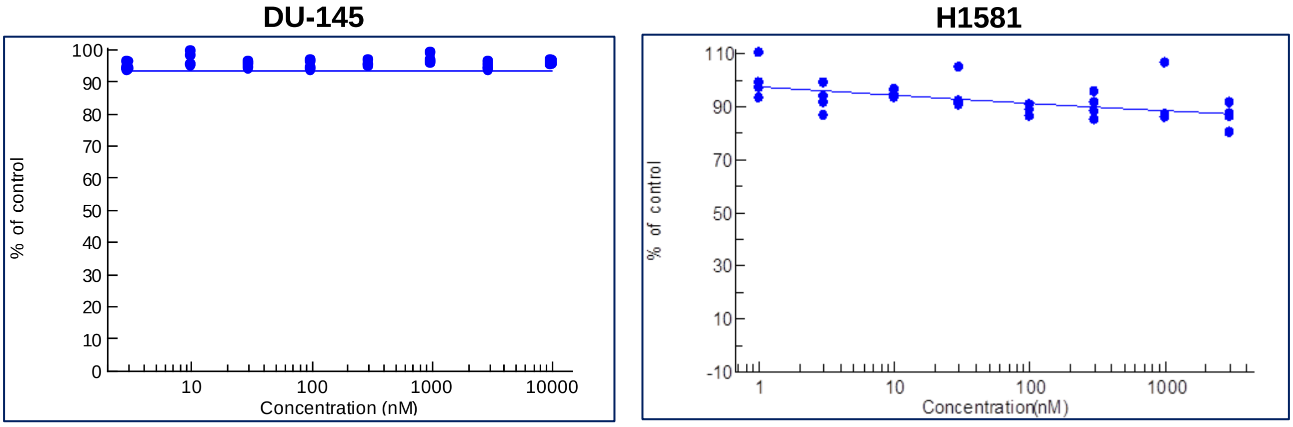

The effect of ODM-201 on the growth of AR-negative cell lines. DU-145 cells (an AR-negative prostate cancer cell line) or H1581 cells (a non-small cell lung cancer cell line) were treated with increasing concentrations of ODM-201 for 4 days, and cell viability was measured using a WST-1 cell proliferation assay. Cell viability is expressed as percentage of the control (100%).

Supplementary Figure S4

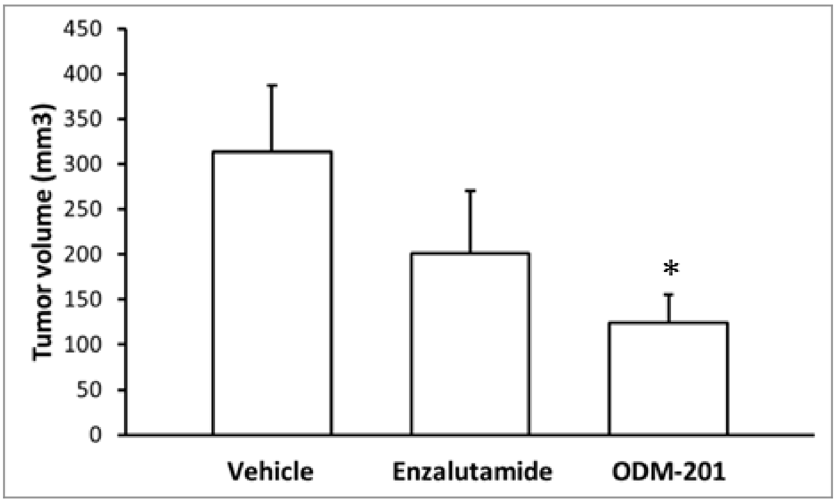

Inhibition of the growth of orthotopic VCaP tumors by ODM-201. For the orthotopic model, 1 x 10^6 VCaP cells were inoculated into the dorsal prostatic lobes of 7-week-old athymic male mice in 20 µL of medium. Tumor growth was monitored with PSA measurements. After 4 weeks, treatments with vehicle, enzalutamide (20 mg/kg, once a day), or ODM-201 (50 mg/kg, twice a day) (n=8) were started and continued for 3 weeks. Mean tumor volumes (mm^3 +SEM) were measured at sacrification. *$p<0.05$ vs. vehicle.

Supplementary Table S1

	**Dose mg/kg**	**AUC_{24} h*ng/ml**	**$T_{1/2}$ h**
**ODM-201**	50 bid	175 000	1.6
**enzalutamide**	20 qd	479 000	18.3

Pharmacokinetic parameters in mice after 7 days oral dosing of ODM-201 or enzalutamide. Based on this exposure data the dose of enzalutamide of 20 mg/kg was selected for *in vivo* efficacy study. Dosing (bid or qd) was selected based on the half-lives ($T_{1/2}$). AUC_{24}=area under the curve at 24 hours. Qd=once daily, bid=twice daily.

Supplementary Table S2

Group	Animal	BW (g) before treatment	Change in body weight (%) at different treatment days (d)					
			**d 3**	**d 9**	**d 16**	**d 23**	**d 30**	**d 37**
ORX	1	22.7	-3.1	-0.4	4.8	0.0	0.4	2.2
ORX	2	23.0	-0.9	0.4	6.5	1.7	3.5*	
ORX	3	19.0	0.0	3.7	5.8	3.2	3.2	5.3
ORX	4	23.4	0.0	0.9	6.4	1.3	1.3*	
ORX	5	21.6	0.0	1.9	6.5	-0.5	-4.6*	
ORX	6	24.5	2.4	3.3	7.8	6.5	-9.8*	
ORX	7	19.8	-1.8	0.0	-0.8	-1.5	-4.5*	
ORX	8	22.2	-1.8	3.2	4.1	-0.9	0.5	0.9
ORX+ enzalutamide	1	23.2	0.0	1.7	3.0	-0.9	-0.9	1.3
ORX+ enzalutamide	2	19.5	0.5	0.5	8.7	1.0	2.1	5.1
ORX+ enzalutamide	3	22.7	-6.2	0.4	1.8	-4.8	-4.0	-0.9
ORX+ enzalutamide	4	22.2	-2.7	3.6	1.8	-1.4	-2.7	0.5
ORX+ enzalutamide	5	16.9	-0.6	0.6	1.2	1.8	3.0	3.6
ORX+ enzalutamide	6	22.9	0.0	1.3	2.6	-2.2	0.9	3.5
ORX+ enzalutamide	7	20.7	-0.5	2.9	6.8	-2.9	1.9	7.2
ORX+ enzalutamide	8	19.8	-2.0	1.5	5.6	-3.0	-2.0	0.5
ORX+ enzalutamide	9	23.8	1.3	1.7	5.5*			
ORX+ ODM-201(qd)	1	23.8	-2.5	-1.7	2.5	-1.3	0.0	-4.6
ORX+ ODM-201(qd)	2	20.1	9.0	7.5	9.5	6.0	1.5	5.0
ORX+ ODM-201(qd)	3	23.2	-1.3	2.2	-1.3	-8.2	-3.9	-6.5
ORX+ ODM-201(qd)	4	19.3	-2.5	3.5	3.0	-1,0	2.5	1.5
ORX+ ODM-201(qd)	5	20.4	-2.0	6.9	14.7	4.4	8.3	6.9
ORX+ ODM-201(qd)	6	24.4	-2.9	-4.9	-5.7	-4.9	-4.1	-7.8
ORX+ ODM-201(qd)	7	20.3	-0.5	3.9	6.9	-0.5	2.0	2.5
ORX+ ODM-201(qd)	8	22.2	-2.3	1.8	0.5	0.9	1.8	1.8
ORX+ ODM-201(qd)	9	23.6	-3.8	-0.8	3.8	-0.4	-0.4	-0.8
ORX+ ODM-201(qd)	10	23.5	-4.7	-7.7	1.3*			
ORX+ ODM-201(bid)	1	23.7	-0.4	-2.5	1.7	-1.7	0.8	0.4
ORX+ ODM-201(bid)	2	24.6	0.0	-9.8	-4.9	-6.5	-6.1*	
ORX+ ODM-201(bid)	3	21.3	-2.8	-0.9	5.6	-2.8	-3.8	-6.1
ORX+ ODM-201(bid)	4	21.1	-1.9	-0.5	3.3	0.5	3.8	-0.5
ORX+ ODM-201(bid)	5	22.3	-1.3	-4.5	0.4	-5.4	-3.6	-6.7
ORX+ ODM-201(bid)	6	24.6	-4.9	-6.9	-4.5	-11.4	-1.6	-1.2
ORX+ ODM-201(bid)	7	20.8	-2.9	-1.9	-1.0	-7.2	-3.4	-6.3
ORX+ ODM-201(bid)	8	24.0	-2,9	-2.1	-†	-†	-1.3*	

The effect of treatments on the body weight of mice bearing subcutaneous VCaP xenograft tumors. Changes (%) in body weights (BW) of castrated (ORX) nude mice with subcutaneous VCaP tumors compared to the BW before the treatments were calculated during the oral treatment with vehicle, enzalutamide (20 mg/kg, qd), or ODM-201 (50 mg/kg, qd or bid) for 37 days (n=8-10). *The mouse had to be euthanised before the end of the experiment due to a large tumor. †Data missing. Qd=once daily, bid=twice daily.

Supplementary Data 1

Description of the synthesis of ODM-201 and ORM-15341

2- Chloro-4-(1-(tetrahydro-2H-pyran-2-yl)-1H-pyrazol-5-yl)benzonitrile

1-(tetrahydro-2H-pyran-2-yl)-5-(4,4,5,5-tetramethyl-1,3,2-dioxaborolan-2-yl)-1H-pyrazole (6.5 g; 23.28 mmol) and 4-bromo-2-chlorobenzonitrile (4 g; 18.48 mmol) were dissolved in THF (65 ml). To this mixture bis(triphenylphosphine)palladium(II) chloride (0.65 g; 0.92 mmol), sodium carbonate (4.7 g; 44.3 mmol) and 18 ml of water were added and the reaction mixture was stirred at 35 ºC for 2.5 h. The solvents were distilled to almost dryness and water (48 ml) was added. After 30 min of stirring the precipitated product was filtered and 32 ml of ethanol was added to the precipitation. The suspension was stirred for 15 min at RT and 30 min at -10 ºC before filtering to give 3.7 g of the product. ^{1}H-NMR (400MHz; d6-DMSO): δ 1.63-1.54 (m, 3H), 1.84-1.80 (m, 1H), 1.97-1.94 (m, 1H), 2.39-2.35 (m, 1H), 3.63-3.57 (m, 1H), 3.99 (m, 1H), 5.32-5.27 (m, 1H), 6.72 (d, 1H), 7.65 (d, 1H), 7.72 (m, 1H), 7.92 (d, 1H), 8.14 (d, 1H).

2- Chloro- 4-(1H-pyrazol-5-yl)benzonitrile

2- Chloro-4-(1-(tetrahydro-2H-pyran-2-yl)-1H-pyrazol-5-yl)benzonitrile (3.67 g; 12.75 mmol) was added to 8 ml of ethanol under nitrogen atmosphere. 15.5 ml of ~10 % HCl (g) in EtOH was slowly added and the temperature was raised to 30 ºC where the mixture was stirred for 1 h. The temperature was then lowered to -10 ºC and the mixture was again stirred for 30 min after which the product was precipitated as its HCl salt and was filtered and washed twice with 2 ml of ethanol. The product was dried in vacuo at +40ºC. Yield 2.8 g. 2- Chloro- 4-(1H-pyrazol-5-yl)benzonitrile hydrochloride (2.8 g; 11.47 mmol) was added to a mixture of 8 ml of water and 14 ml of MeOH under nitrogen atmosphere. To this 50 % sodium hydroxide (1.5 ml; 28.7 mmol) was added keeping the temperature under 25 ºC during the addition. The mixture was stirred for 2 h, the precipitate

filtered and washed twice with 3 ml of lukewarm water. The product was dried in vacuo at +40 ºC. Yield 1.97 g. ^{1}H-NMR (400MHz; d6-DMSO): δ 6.99 (t, 1H), 7.89 (m, 1H), 7.99 (d, 2H), 8.15 (s, 1H), 13.27 (s, 1H).

(S)-4-(1-(2-aminopropyl)-1H-pyrazol-3-yl)-2-chlorobenzonitrile

2- Chloro- 4-(1H-pyrazol-3-yl)benzonitrile (4.00g; 19.64 mmol), *(S)-tert*-butyl-1-hydroxypropan-2-yl carbamate (3,79 g; 21.61 mmol) and triphenylphosphine were dissolved in dry THF under nitrogen athmosphere and stirred. Diisopropylazodicarboxylate (7.74 ml; 39.3 mmol) was added dropwise and the reaction flask was cooled by ice bath. The reaction was stirred at RT overnight (18 h) and evaporated to dryness. For Boc deprotection 200 ml of 10 % HCl/EtOH solution was added to the evaporation residue, stirred for 20 h at RT and evaporated to dryness. 100 ml of water was added to the evaporation residue and washed with 3 x 120ml of DCM to remove reactant residues. pH of water phase was adjusted to ~12 by addition of 2 M NaOH, washed with 3 x 80 ml of DCM and organic phase dried over Na_2SO_4. Organic phase was filtered and evaporated to give 2.605 g of the title compound.

(S)-3-acetyl-N-(1-(3-(3-chloro-4-cyanophenyl)-1H-pyrazol-1-yl)propan-2-yl)-1H-pyrazole-5-carboxamide ORM-15341

3-Acetyl-1H-pyrazole-5-carboxylic acid (0.59 g; 3.84 mmol) and DIPEA (1.0 ml; 5.75 mmol) were dissolved in 4 ml of dry DCM. Anhydrous HOBt (0.78 g; 5.75 mmol) and EDCI (1.10 g; 5.75 mmol) were added at RT. (S)-4-(1-(2-aminopropyl)-1H-pyrazol-3-yl)-2-chlorobenzonitrile (1.00 g; 3.84 mmol) was dissolved in 4 ml of DCM and the reaction was stirred for overnight at RT. 40 ml of DCM was added and organic layer washed with 3 x 15 ml of water. Combined water phases were washed with 2 x 20 ml of DCM. Both organic phases were dried over Na_2SO_4, filtered and evaporated to dryness. Both crude product fractions were combined and purified by CombiFlash (2 % MeOH in DCM). Product fractions were combined and evaporated to give 497mg of product. ^{1}H-

NMR (400MHz; d6-DMSO): δ 1.16 (d, 3H, J=6.7Hz), 2.49 (s, 3H), 4.31 (m, 2H), 4.46 (sept, 1H, J=6.7 Hz), 6.93 (d, 1H, J=2.4 Hz), 7.31 (s, 1H), 7.81 (d, 1H, J=2.4Hz), 7.92 (d, 1H, J=7.9Hz), 7.97 (d, 1H, J=8.1Hz), 8.03 (d, 1H, J=1.3Hz), 8.48 (d, 1H, J=8.5Hz), 14.16 (s, 1H).

N-((S)-1-(3-(3-chloro-4-cyanophenyl)-1H-pyrazol-1-yl)-propan-2-yl)-3/5-(1-hydroxyethyl)-1H-pyrazole-5/3-carboxamide ODM-201

(*S*)-3-acetyl-N-(1-(3-(3-chloro-4-cyanophenyl)-1H-pyrazol-1-yl)propan-2-yl)-1H-pyrazole-5-carboxamide (100 mg; 0.25 mmol) and 5ml of EtOH were charged to the reaction flask and $NaBH_4$ (19 mg; 0.5 mmol) was added slowly as EtOH suspension. The reaction was stirred overnight to completion followed with addition 0.5 ml of water and 1ml of 0.5M HCl dropwise. The solution was evaporated to dryness, 20 ml of dichloromethane was added and washed with 10 ml of 1 M $NaHCO_3$ and 10 ml of water followed with drying over Na_2SO_4. After filtration and evaporation 76 mg of ODM-201 was obtained. 1H-NMR (400MHz; d6-DMSO): δ 1.11 (d, 3H), 1.38 (d, 3H), 4.22-4.48 (m, 3H), 4.74-4.84 (m, 1H), 4.41 (d, 1H), 6.40 (s, 1H), 6.94 (d, 1H), 7.81 (d, 1H), 7.92-8.05 (m, 2H), 8.09 (d, 1H), 8.20 (d, 1H), 13.04 (s, 1H).
